# Acute Oral Chromium Exposure Resulting in Ulcerative Gastritis and Perforated Ulcers in Swine

**DOI:** 10.3390/ani14010063

**Published:** 2023-12-23

**Authors:** Fernanda Felicetti Perosa, Anderson Hentz Gris, Manoela Marchezan Piva, Jean Carlo Olivo Menegatt, Claiton Ismael Schwertz, Paola Sônego, Tatiane Terumi Negrao Watanabe, Saulo Petinatti Pavarini, David Driemeier, Welden Panziera

**Affiliations:** 1 Setor de Patologia Veterinária, Faculdade de Medicina Veterinária, Universidade Federal do Rio Grande do Sul (UFRGS), Av. Bento Gonçalves, 9090, Porto Alegre 91540-000, RS, Brazil; anderson_gris@hotmail.com.br (A.H.G.); manoela.marchezan@gmail.com (M.M.P.); menegattjean2@gmail.com (J.C.O.M.); claiton.schwertz@outlook.com (C.I.S.); sonegopaola@gmail.com (P.S.); sauloppvet@yahoo.com.br (S.P.P.); ddriemeier@gmail.com (D.D.); weldenpanziera@yahoo.com.br (W.P.); 2 Laboratório de Patologia Veterinária, Universidade Federal do Paraná—Setor Palotina (UFPR), Rua Pioneiro, 2153, Palotina 85950-000, PR, Brazil; 3 Inata Biológicos, BR-365, KM-615, Uberlândia 38407-180, MG, Brazil; 4 Department of Population Health and Pathobiology, College of Veterinary Medicine, North Carolina State University, Raleigh, NC 27606, USA; tati.terumi@yahoo.com.br; 5 Antech Diagnostics, West Olympic Blvd, Los Angeles, CA 90064, USA

**Keywords:** hexavalent chromium, poisoning, heavy metals, corrosive lesions, gastric ulcer, necropsy

## Abstract

**Simple Summary:**

Chromium is a heavy metal often utilized in industrial applications in its hexavalent form, which is particularly toxic. It also serves as a trace mineral to organisms in its trivalent form. In humans, accidental or intentional ingestion of high amounts of hexavalent chromium leads to corrosive lesions in the gastrointestinal tract, potentially resulting in death due to extensive areas of necrosis and hemorrhage in the stomach and intestines. In a swine farm, pigs housed in bedding of pine wood shavings containing chromium died secondarily to ulcerative gastritis with subsequent perforation of the glandular stomach. This study marks the first description of natural acute oral chromium poisoning in animals.

**Abstract:**

Heavy metal poisoning poses a challenge in diagnostic practices and environmental safety. This study describes the epidemiological, clinical, and pathological aspects of a chromium (Cr) poisoning outbreak in growing/finishing pigs housed in pens with bedding of pine wood shavings containing Cr. A visit to the affected farm was conducted. Epidemiological data were collected, and necropsy and histopathological examinations and heavy metal quantifications were performed. Up to 30% of the animals from the affected pens displayed clinical signs 48 h after housing, characterized by apathy, rigid gait, distended abdomen, pain to abdominal palpation, fever, vomiting, and skin cyanosis. The lethality rate reached 76.6%. Main postmortem findings consisted of ulcerative gastritis with perforation of the glandular stomach in all necropsied swine. Heavy metal analysis revealed a higher concentration of Cr in the bedding of the affected pens, along with elevated levels of Cr in the livers of the affected swine. Given that Cr is a known cause of poisoning in humans (with acute oral exposure resulting in corrosive lesions in the gastrointestinal tract), this study marks the first diagnosis of acute oral natural Cr poisoning in animals. This diagnosis was established through the association of epidemiological, pathological, and heavy metal quantification data.

## 1. Introduction

Chromium (Cr) is a metallic element first discovered in 1797 by the French chemist L. N. Vauquelin, who identified it in a Siberian mineral now known as crocoite. Its name, derived from the Greek word “chroma” meaning color, reflects the variety of colors found in its compounds [1]. Since its initial identification, Cr and its compounds have emerged as among the world’s most strategically important materials, with extensive application in metalworking and chemical industries. Cr exists in various oxidation states, with trivalent (III) and hexavalent (VI) being the most prevalent [1,2]. While Cr(III) is an essential dietary mineral [3], Cr(VI) can also occur in the natural environment, though it is a suspected carcinogen and a potential contaminant in soil and water [2].

Occupational Cr poisoning is a concern in human medicine, particularly for workers exposed to Cr(VI) [4]. Studies have shown that Cr(VI) undergoes conversion to Cr(III), primarily in the acidic environment of the stomach but also in the bloodstream [5,6,7]. This conversion generates free radicals that can form complexes with intracellular targets, resulting in DNA damage and playing a crucial role in the observed carcinogenicity associated with chronic exposures [8]. When in its hexavalent state (VI), Cr is highly corrosive. In acute poisonings, contact with the skin and airways or ingestion can lead to severe and potentially fatal tissue irritation [2]. Accidental or intentional ingestions of Cr(VI) by children and adults have been documented, causing necrosis and massive hemorrhage of the gastrointestinal tract. Main clinical signs in these cases included abdominal pain, vomiting, and bloody diarrhea [9,10,11,12].

In animal studies, the application of potassium dichromate (Cr(VI)) to the oral mucosa at a dosage of 30 mg/kg or through intragastric administration at 5 mg/kg was found to induce local corrosive lesion associated with renal failure leading to death [13]. Other studies have shown that Cr(VI) exhibited greater toxicity to the liver, brain, kidney, and myocardium compared with Cr(III) in rats following intraperitoneal administration (2 mg/kg daily for 3–6 weeks) [13]. Similarly, in rats, Cr(III) exhibited its highest levels of accumulation in the liver and kidneys, followed by the testes, brain, and blood, after intraperitoneal administration at doses of 1, 2, and 3 mg/kg [14].

In swine, gastric ulcers affecting the *pars esophagea* (aglandular stomach) are a significant cause of reduction in productivity and increase in mortality, particularly in growing/finishing pigs and in sows [15,16,17]. Several risk factors contributing to the development of *pars esophagea* ulcers are discussed, with the primary factors being associated with particle size and composition of the diet, interruptions in feed intake, stress, the use of non-steroidal anti-inflammatories, and infectious diseases [15,18]. However, these ulcers typically do not extend to the glandular stomach, as the mucosa in this area produces mucous that covers the lumen and protects it against the stomach acid [15]. Although poisonings in swine production systems are uncommon [19], cases of gastritis affecting the glandular stomach have been reported as a consequence of exposure to toxic compounds such as arsenic, thallium, formalin, bronopol, and phosphatic fertilizers [15].

This present study aims to describe the epidemiological, clinical, and pathological aspects of an outbreak of ulcerative gastritis with perforated ulcers in the glandular stomach in growing/finishing pigs housed in pens with bedding of wood shavings containing Cr.

## 2. Materials and Methods

### 2.1. Epidemiological Data and Clinical Signs

A visit to the pig farm was conducted during the disease outbreak. Epidemiological data, including composition of the pens’ bedding, date and conditions of housing, vaccination scheme, the onset of the clinical outbreak, clinical progression, treatment performed, and morbidity, mortality, and lethality rates, were recorded. Additionally, animals that were still affected during the visit were assessed to document their clinical signs.

### 2.2. Postmortem Examination

Postmortem examinations were performed on four animals. A systematic necropsy, involving the examination of all organs, was carried out. Major organs, including liver, gallbladder, stomach, spleen, kidneys, urinary bladder, lymph nodes, heart, lungs, central nervous system, and intestines, were sampled. Tissues were then fixed in 10% formaldehyde solution, followed by histologic processing and staining with hematoxylin and eosin (HE) for microscopic examination.

### 2.3. Heavy Metal Quantification

Following the suspected diagnosis of poisoning, tissue sections from the liver, kidneys, and skeletal muscles of swine 1 and 2, along with samples of the bedding (pine and eucalyptus wood shavings), were collected for heavy metals’ quantification using atomic absorption spectroscopy. Samples of liver and bedding of pine wood shavings were analyzed for Cr (Atomic Absorption Spectrophotometer, Zeenit 700 BU, Analytik Jena, Thuringia, Germany, graphite furnace with Zeeman correction; minimum detectable value of 0.05 mg/kg) [20], copper (Cu) (Atomic Absorption Spectrophotometer, G84324, Agilent, California, USA; detection by flame) [21], and arsenic (As) (Atomic Absorption Spectrophotometer, Zeenit 700 BU, Hydride Generation: HS60 Modular, Analytik Jena, Thuringia, Germany; minimum detectable value of 0.06 mg/kg) [22]. Meanwhile, samples of the kidneys and skeletal muscles, as well as the bedding of eucalyptus wood shavings, were assessed solely for Cr (Atomic Absorption Spectrophotometer, Zeenit 700 BU, Analytik Jena, Thuringia, Germany; graphite furnace with Zeeman correction; minimum detectable value of 0.05 mg/kg) [20]. Additionally, a sample of liver from a swine that died from a different condition while housed in a non-affected pen (with bedding of eucalyptus wood shavings) was submitted for Cr analysis to serve as a control. The analyses were conducted based on the wet weight of the samples.

## 3. Results

### 3.1. Epidemiological Data and Clinical Signs

A disease outbreak was recorded in a growing/finishing pig farm located in the municipality of Antônio Prado (28°51′28″ S 51°16′58″ O), Rio Grande do Sul, Brazil. The farm consisted of multiple barns, with a total capacity for 2700 swine. In one of the barns, 5 days prior to the visit, 500 swine of a single origin, aged 65 days, were housed in five different pens, averaging 100 swine per pen. The animals were vaccinated in the nursery with commercial vaccines for *Mycoplasma hyopneumoniae* and porcine circovirus type 2, as well as autogenous vaccines against *Glaesserella parasuis*, *Pasteurella multocida*, and *Salmonella* spp.

The bedding in this barn consisted of wood shavings, with three pens using eucalyptus (*Eucalyptus* sp.) wood shavings (300/500 pigs housed) and the remaining two pens using pine (*Pinus elliottii*) wood shavings (200/500 pigs housed). The farm usually performed a 15-day sanitary gap. At the end of each batch, the bedding was removed, and quicklime and fresh wood shavings were added on top.

**Figure 1:**
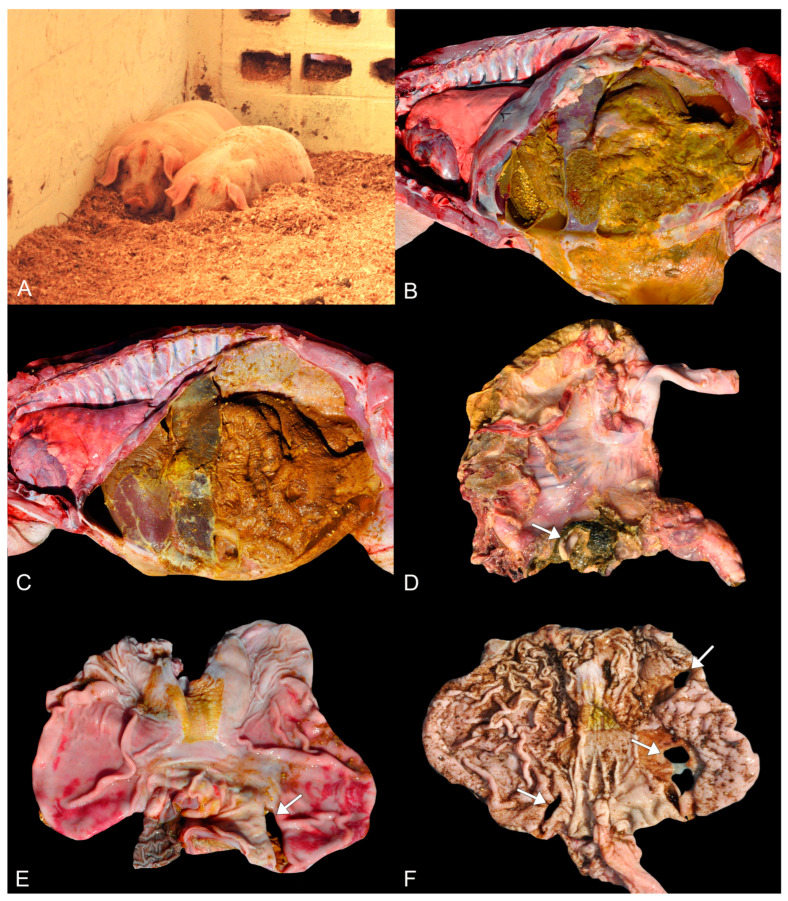
Clinical and macroscopical findings of perforated ulcerative gastritis in swine associated with acute oral chromium exposure. (**A**) Two pigs on the bedding of pine wood shavings in sternal recumbency and lethargy. (**B**,**C**) Marked peritonitis characterized by deposition of gastric contents and fibrin on the serosa of the abdominal organs. Swine 3 and 1, respectively. (**D**) Stomach. A focal area, nearly 2 cm in diameter, of perforation (arrow) is observed in the fundus portion of the stomach wall. The perforation has slightly raised and firm edges. Additionally, there is deposition of yellow to green fibrillary material on the gastric serosa. Swine 2. (**E**) Stomach. Glandular mucosa from the fundus region presenting a focal area of nearly 3 cm in diameter of perforation with slightly elevated and firm edges (arrow). The remaining glandular mucosa shows multifocal to coalescent areas of moderate reddening (hyperemia). Hyperparakeratosis of the *pars esophagea* (aglandular stomach) is also present. Swine 1. (**F**) Stomach. In the glandular mucosa, three areas of perforation are noted (arrows), each measuring from 2 to 3 cm in diameter, with two in the antropyloric region and one in the body of the stomach. The remaining mucosa exhibits a significant amount of millimetric multifocal to coalescent areas slightly depressed and darkened (erosions). The *pars esophagea* also exhibits slight hyperparakeratosis. Swine 4.

In the morning, 2 days after the 500 swine were housed (48 h), about 60 pigs housed in the two pens using pine (*Pinus elliottii*) wood shavings began displaying clinical signs of apathy (Figure 1A), rigid gait, distended abdomen, pain to abdominal palpation, fever, and frequent vomiting. Despite treatment with antitoxic (hepatoprotective drugs and multivitamin complex) and dexamethasone administered via water, there was no clinical improvement. By the afternoon of the same day (56 h after housing), 46 pigs had succumbed to death, typically found in sternal recumbence with cyanosis of the skin. The pigs were then moved to a pen with eucalyptus bedding, and no further animals started presenting clinical signs.

At the time of our visit, three days after the onset of clinical signs, 46 animals died. All deaths occurred only in pens with bedding of pine wood shavings. Thus, morbidity and mortality rates in the two affected pens were 30% (60/200 pigs) and 23% (46/200 pigs), respectively. The lethality rate reached 76.6% (46/60 pigs).

According to the farm workers, and as was also noticeable during the visit, the swine habitually ingested considerable amounts of the bedding material. All pigs at the farm were fed with feed produced on the farm composed of corn, soy, barley, a commercial premix (commercial product composed of a mixture of vitamins and minerals), tiamulin, amoxicillin, and colistin. Feed was supplied associated with whey ad libitum through automatic feeders, and water was also provided ad libitum.

### 3.2. Postmortem Examination

Four swine (67 days) were necropsied during the visit (animals 1, 2, 3, and 4), comprising three males (1, 2, and 4) and one female (3). All of them had good body condition and displayed skin cyanosis. Swine 1, 3, and 4 had congested mucous membranes, while animal 2 had pale mucous membranes.

Upon internal examination, all animals presented a large amount of liquid in the abdominal cavity, ranging from brown (animal 1), brown-greenish (animal 2), yellow (animal 3), to brown-reddish (animal 4). This liquid content was associated with the presence of gastric content and marked deposition of fibrillary yellow material (fibrin) on the serosa of the abdominal organs (Figure 1B,C). Small fragments of wood shavings were also sometimes visible amidst the extravasated gastric contents.

In the glandular stomach, mainly in the fundus portion, from swine 1, 2, and 3, there was a focal area of complete perforation (perforated ulcers) ranging from 2 to 4 cm in diameter. The perforated ulcers exhibited firm and raised edges (Figure 1D,E). The remaining gastric mucosa displayed multifocal and moderate areas of hyperemia. However, animal 4 had three perforations, each measuring 2 to 3 cm in diameter, with two of them in the antropyloric region and the other in the body of the stomach. The remaining glandular mucosa from swine 4 displayed multifocal to coalescent slightly depressed and darkened millimetric areas (erosions) (Figure 1F).

In the cardia from all animals, diffuse and moderate hyperkeratosis was also observed, which extended to the esophagus in animals 2 and 3. Animal 2 also had multifocal areas of erosion in the esophageal mucosa. Furthermore, all animals exhibited diffuse reddening of the intestines, along with enhanced blood vessels on the serosa. Macroscopic evaluation of the remaining organs was unremarkable.

**Figure 2:**
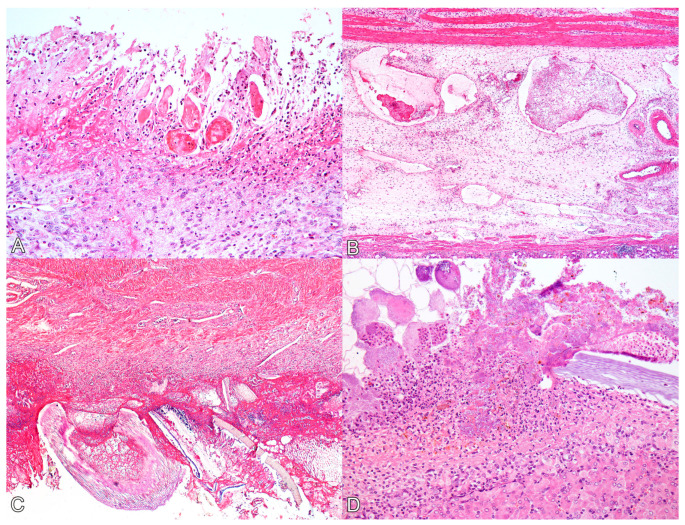
Histological lesions of perforated ulcerative gastritis in swine associated with acute oral chromium exposure. (**A**) Stomach. There is diffuse necrosis of the glandular mucosa, characterized by loss of tissue and cellular architecture and associated with hyperemia, neutrophil infiltration, and fibrin deposition. Swine 3. HE, 200×. (**B**) Stomach. The submucosa exhibits marked edema, and lymphatic vessels appear ectasic and filled with fibrin thrombi. Swine 3. HE, 100×. (**C**) Stomach. On the serosal surface, there is marked fibrin deposition, infiltration of neutrophils, bacterial myriad, and fragments of plant fibers from the gastric content. Swine 4. HE, 100×. (**D**) Liver. The Glisson’s capsule shows marked fibrin deposition, neutrophil infiltration, and bacterial myriad. A plant fiber is also noted amidst the lesion. Swine 4. HE, 200×.

Histologically, the main lesions were observed in the glandular stomach. In the mucosa of the glandular stomach of all pigs, there was marked ulceration (Figure 2A), characterized by necrosis of the epithelium and tissue discontinuity, along with pronounced inflammatory infiltrate of neutrophils, deposition of fibrin, and coccobacillary bacterial myriad. The inflammatory infiltrate extended into the submucosa and muscular layers, where it was associated with severe edema and vascular thrombosis (Figure 2B). Additionally, multiple fibrin thrombi were observed within lymphatic vessels in the submucosa. The mucosa adjacent to the ulcerations exhibited marked congestion. In the mucosa of the *pars esophagea* (aglandular stomach) and esophagus, all animals displayed moderate and diffuse parakeratotic hyperkeratosis, accompanied by multifocal areas of erosion in the cardia (swine 1 and 4) and esophagus (swine 2 and 3).

In swine 1, 3, and 4, the duodenum exhibited multifocal areas of moderate erosion of the epithelium, characterized by necrosis of enterocytes and inflammatory infiltration of lymphocytes, plasma cells, and histiocytes in the lamina propria.

On the serosal surfaces of the stomach (Figure 2C), small and large intestines, and urinary bladder (as well as in the hepatic (Figure 2D) and splenic capsules), there was marked fibrin deposition, along with abundant plant fibers. Additionally, inflammatory infiltration of neutrophils and coccobacillary bacterial myriad were noted.

### 3.3. Heavy Metal Quantification

Results of heavy metal quantification from the liver, kidney, and skeletal muscles of affected swine, as well as from the liver of the control, and the pens’ bedding are depicted in Table 1.

## 4. Discussion

In the present study, the diagnosis of poisoning was strongly suspected due to the macroscopic and microscopic findings of ulcerative gastritis with perforated ulcers in the glandular stomach of all necropsied swine affected by the outbreak. Considering the history of these swine ingesting the wood shavings used in the bedding, there was a strong suspicion that the animals had inadvertently consumed a toxin present in the bedding material. Therefore, an investigation was carried out to test the bedding for the presence of chemical substances known to be caustic, aiming to identify any potentially hazardous components that could cause ulcerative lesions. Remarkably, three heavy metals known to cause lesions in the gastrointestinal mucosa were examined: As, Cu, and Cr [14,25,26,27].

As, Cu, and Cr are components present in the mixture known as chromated copper arsenate (CCA), a widely used wood preservative pesticide. CCA is classified into three types: A, B, and C. Notably, Cr(VI) constitutes a significant portion of CCA, accounting for 65.5%, 35.3%, and 47.5% of the total composition of CCA types A, B, and C, respectively [28]. In the present report, a significant concentration of Cr was identified in the bedding of pine wood shavings, indicating that the wood could have been previously treated with CCA or a similar product containing Cr. This concentration was higher in the pine bedding when compared with the bedding of eucalyptus wood shavings, coinciding with the observation that all affected animals were housed in pens with pine bedding. Moreover, the elevated levels of Cr found in the livers of the affected swine, when compared to reference values, and the values of Cu and As being within the limits, further support the diagnosis of Cr poisoning [23,24,29].

Cases of Cr-related poisoning in humans, resulting from accidental or intentional ingestion, have been documented. Cr(VI) is highly corrosive to the gastrointestinal tract, and ingestion of extremely high doses of Cr(VI) can lead to hemorrhagic gastroenteritis [30]. A 35-year-old woman who ingested chromic acid experienced massive gastrointestinal hemorrhage, followed by severe acidosis, acute renal failure, and hepatic injury. The woman died 12 h after the ingestion, and postmortem analysis revealed extensive necrosis of the entire digestive mucous membrane [14]. Another case involved a 17-year-old male who ingested 29 mg of Cr(VI) per kg of body weight in a suicide attempt. He died 14 h after ingestion, displaying caustic burns in the stomach and duodenum, as well as gastrointestinal hemorrhage [12]. Reports of infants dying after ingestion of unknown amounts of Cr components have described necrosis of the gastrointestinal tract as the cause of death [11,13]. The findings from acute oral exposure to Cr in humans mirror the lesions observed in the affected swine during this outbreak. The main lesions reported here were characterized by stomach perforation in all animals, secondary to ulcerative gastritis. Peritonitis was observed in all animals, resulting from the perforation of the stomach and subsequent leakage of gastric contents into the abdominal cavity. These perforated ulcers and the secondary peritonitis provided an explanation for the abdominal distension and pain detected during abdominal palpations in the affected animals within the herd.

In experimental studies conducted on rats, oral exposure to Cr(VI) resulted in irritation of the gastrointestinal tract. Rats subjected to a lethal gavage dose of potassium dichromate of 130 mg Cr(VI)/kg demonstrated gastrointestinal hemorrhage [31]. In parallel with these findings, our cases presented striking histological findings characterized by necrotic lesions in the mucosa of the glandular stomach, including transmural ulceration and fibrinosuppurative inflammation. Furthermore, the aglandular stomach, which is the region frequently affected by ulcers in swine [15,18], did not exhibit any lesions, despite the presence of parakeratosis. This may imply that the lesions observed in the glandular stomach were likely caused by direct contact with the toxic agent.

In contrast, rats subjected to longer periods of exposure exhibited a more histiocytic inflammatory pattern of lesion when ingesting sodium dichromate dihydrate (SDD) [32,33]. SDD is a Cr(VI) compound found in drinking water supplies as a contaminant from industrial processes [4]. In an experiment, ingestion of doses higher than 170 mg/L of SDD in drinking water over 90 days resulted in villous atrophy, apoptosis, crypt cell hyperplasia, and histiocytic infiltration [33]. Meanwhile, in other studies on rats and mice over four weeks, the incidence of histiocytic cellular infiltration increased in the duodenum when the animals were exposed to 125 mg/L of SDD. Additionally, ulceration, regenerative epithelial hyperplasia, and squamous epithelial metaplasia occurred in the glandular stomach of rats exposed to 1000 mg/L of SDD [32]. The swine in this report also displayed erosive lesions of the duodenal mucosa along with histiocytic inflammatory infiltration. However, these animals underwent a more acute clinical exposure period when compared with the aforementioned studies in rats. Unfortunately, it was not possible to estimate the quantity of pine wood shavings ingested by each animal and, consequently, their intake of Cr.

Heavy metal analysis of the affected swine’s organs revealed a notable increase in Cr concentration, particularly in the livers of the tested animals. Reference values for Cr concentration in swine liver exhibit a wide range. Studies have reported mean values ranging from 0.01 mg/kg to 0.12 mg/kg in the livers of slaughtered pigs [23,24]. When taking into consideration our findings, where swine 1 and 2 exhibited Cr concentrations of 1 mg/kg and 0.56 mg/kg, respectively, these values were significantly elevated. In swine 1, the concentration was at least 8.3 times higher than the findings of Lopez-Afonso et al. [24], and up to 100 times higher when compared with the results of Jorhem et al. [23] for normal levels. In cases of Cr exposure in humans, it is established that the absorbed Cr distributes throughout nearly all tissues, with the liver and kidneys demonstrating the highest concentrations [30]. However, the concentrations of Cr in the kidneys of the tested animals were below the minimum detection limit of the examination. We believe this was linked to the shorter clinical course of our cases. Furthermore, determining the dosage of gastric content was no longer viable once it had leaked into the abdominal cavity precluding sampling.

In swine, gastroesophageal ulceration affecting the *pars esophagea* is one of the most important conditions affecting the stomach [15]. While the causes of gastric ulceration remain incompletely understood, several risk factors are discussed. These include diets rich in corn and wheat, fine particle size, interruptions in feed intake, acute infectious diseases leading to reduced appetite and increased histamine levels, the use of non-steroidal anti-inflammatories, as well as *Helicobacter*-like organism infections, as suggested in experimental studies [15,18]. Ulceration of the *pars esophagea* typically has the highest ulceration rate in growing/finishing pigs and in sows, with mortality rates ranging from 1–2% [18]. However, recent studies have shown mortality rates of 15.4% and 9.9% in growing/finishing pigs and sows, respectively [16,17]. Yet, in the present report, the mortality rate was significantly higher, up to 23%, and the clinical, pathological, and epidemiological findings differed greatly from those seen in sporadic cases of gastric ulcers, such as those reported in the aforementioned studies [16,17].

Lesions associated with ulceration of the *pars esophagea* rarely extend into the contiguous esophagus or the glandular region of the stomach [15]. Glandular stomach ulcers represent less than 1% of all diagnosed ulcers in swine, and their main causes are infectious diseases such as circovirus, transmissible gastroenteritis, salmonellosis, classic swine fever, and endoparasitosis [18]. In the necropsied swine from this outbreak, the ulcers were limited to the glandular mucosa, differing from the more prevalent occurrence in the aglandular mucosa (*pars esophagea*). Furthermore, none of the animals presented macroscopic or microscopic findings compatible with the above-mentioned infectious disease, known to sporadically result in ulceration of the glandular stomach. Moreover, gastritis in the glandular mucosa can occur secondarily to fungal infections by *Rhizopus*, *Absidia*, *Mucor*, and *Aspergillus* species. This type of mycotic gastritis is occasionally observed in piglets, typically in association with repeated antibiotic use. Lesions present as multifocal yellowish plaques on the gastric mucosa [15]. In our report, the animals were older, in the growing/finishing phase, and the lesions differed from the pattern observed in cases of mycotic gastritis, thereby ruling out this potential diagnosis.

## 5. Conclusions

To the best of the authors’ knowledge, this is the first report of natural acute oral Cr poisoning in animals. We hypothesize that the affected swine in this outbreak ingested pine wood shavings containing substantial quantities of Cr, culminating in the development of ulcerative gastritis and subsequent perforation of the glandular stomach. In addition, Cr quantification in the livers of two affected animals yielded a significant increase in Cr levels compared with reference values in the literature and from the liver of the control animal. Therefore, acute oral Cr poisoning in pigs should be considered in the differential diagnosis of ulcerative gastritis, especially in cases of ulcers affecting the glandular stomach.

In swine farms employing wood shavings as bedding, caution must be exercised regarding the prior treatment of the wood, especially with preservative pesticides containing Cr(VI), which represents the most toxic and highly corrosive form of Cr. Farm employees in contact with treated wood shavings are at risk of experiencing dermal and respiratory irritation.

## Figures and Tables

**Table 1:** Heavy metal levels in organs of affected swine in an outbreak of perforated gastric ulcer due to chromium poisoning.

Identification	Sample/Tissue *	Heavy Metal (mg/kg)		
		Chromium	Copper	Arsenic
Swine 1	Liver	1	5.45	<0.06
	Kidney	<0.05	-	-
	Skeletal muscle	<0.05	-	-
Swine 2	Liver	0.56	5.05	<0.06
	Kidney	<0.05	-	-
	Skeletal muscle	<0.05	-	-
Control	Liver	<0.05	-	-
Reference value (mean values)	Liver	0.01 [23], 0.12 [24]	9 [23], 14.9 [24]	0.013 [24]
	Kidney	0.01 [23], 0.077 [24]	-	-
	Skeletal muscle	0.014 [23], 0.131 [24]	-	-
Pine bedding		5.75	3.51	<0.06
Eucalyptus bedding		3.46	-	-

* The analyses were conducted based on the wet weight of the samples.

## Data Availability

The data presented in this study are available on request from the corresponding author. The data are not publicly available due to privacy concerns related to the pig farm.
